# Age-related differences in processing speed in children can be explained by heterochronicity of human brain development

**DOI:** 10.1192/j.eurpsy.2021.1300

**Published:** 2021-08-13

**Authors:** S. Kiselev

**Affiliations:** Clinical Psychology, Ural Federal University, Ekaterinburg, Russian Federation

**Keywords:** processing speed, Brain Development, heterochronicity

## Abstract

**Introduction:**

Age-related differences in the processing speed have been observed in a great variety of tasks. In spite of the great amount of researches in this area, we know relatively little about the nature of this developmental tendency.

**Objectives:**

The aim of this study was to assess whether age-related differences in reaction time (RT) can be explained satisfactorily in terms of a global age-related differences in processing speed alone.

**Methods:**

The sample consisted of 48 4-year-olds, 50 5-year-olds, 46 6-year-olds children, and 35 adults. To investigate processing speed in children and adults we used the test battery consisted of three types of RT tasks: simple, discrimination, and choice.

**Results:**

We have revealed clear age-related differences in processing speed not only between children and adults but also between three age groups of children. However, using transformation method proposed by Madden et al. (2001) and Ridderinkhoff & van der Molen (1997) we have revealed that there are not only global age-related differences but also process-specific age-related differences in processing speed. Among children, age-related differences larger than predicted by the global difference hypothesis were evident when tasks required spatial orientation discrimination and stimulus–response rule complexity, but not for response suppression or reversal of stimulus–response contingencies.

**Conclusions:**

It can be assumed that the observed process-specific, age-related differences in processing speed generally can be explained by the principle of heterochronicity of human brain development (Casey et al., 2005).

